# *Plasmodium falciparum* outbreak in native communities of Condorcanqui, Amazonas, Perú

**DOI:** 10.1186/s12936-021-03608-2

**Published:** 2021-02-12

**Authors:** Carla C. Montenegro, T. Pershing Bustamante-Chauca, Cecilia Pajuelo Reyes, Miguel Bernal, Lizandro Gonzales, Rafael Tapia-Limonchi, Juan R. Tejedo, Stella M. Chenet

**Affiliations:** 1 Instituto de Enfermedades Tropicales, Universidad Nacional Toribio Rodríguez de Mendoza (UNTRM), Amazonas, Peru; 2 Dirección Regional de Salud (DIRESA), Amazonas, Peru; 3 Red de Salud Condorcanqui, Amazonas, Peru; 4 Departamento de Biología Molecular E Ingeniería Bioquímica, Universidad Pablo de Olavide (UPO), Seville, Spain; 5 Diabetes and Associated Metabolic Diseases Networking Biomedical Research Centre (CIBERDEM), Madrid, Spain

**Keywords:** Malaria, Peru, Prevalence, Epidemiology, *Plasmodium vivax*, *Plasmodium falciparum*, Asymptomatic malaria, Low parasitaemia, Spatial clustering, Native communities

## Abstract

**Background:**

Malaria remains a serious health threat in the Amazonas Region of Peru and approximately 95% of the cases, mainly *Plasmodium vivax*, are found in native communities of The Rio Santiago District, Condorcanqui Province. In 2019, more than one thousand malaria cases were reported, with an unusual number of *Plasmodium falciparum* autochthonous cases. The present study aims to report this *P. falciparum* outbreak while describing the epidemiology of malaria and the risk factors associated in the native communities of Amazonas, Peru.

**Methods:**

The DIRESA-Amazonas in collaboration with the Condorcanqui Health Network and the Institute of Tropical Diseases of the UNTRM carried out a malaria Active Case Detection (ACD III) between January 31st and February 10th of 2020. A total of 2718 (47.4%) individuals from 21 native communities grouped in eight sanitary districts, were screened for malaria infections. Each participant was screened for malaria using microscopy. Follow-up surveys were conducted for all malaria positive individuals to collect socio-demographic data. Spatial clustering of infection risk was calculated using a generalized linear model (GLM). Analysis of risk considered factors such as gender, age, type of infection, symptomatology, and parasitaemia.

**Results:**

The study suggests that the *P. falciparum* index case was imported from Loreto and later spread to other communities of Rio Santiago during 2019. The ACD III reported 220 (8.1%) malaria cases, 46 *P. falciparum*, 168 *P. vivax* and 6 mixed infections. SaTScan analysis detected a cluster of high infection risk in Middle Rio Santiago and a particular high *P. falciparum* infection risk cluster in Upper Rio Santiago. Interestingly, the evaluation of different risk factors showed significant associations between low parasitaemia and *P. falciparum* asymptomatic cases.

**Conclusion:**

This is the first report of a *P. falciparum* outbreak in native communities of Condorcanqui, Amazonas. Timely identification and treatment of symptomatic and asymptomatic cases are critical to achieve malaria control and possible elimination in this area. However, the current malaria situation in Condorcanqui is uncertain, given that malaria ACD activities have been postponed due to COVID-19.

## Background

**Fig. 1:**
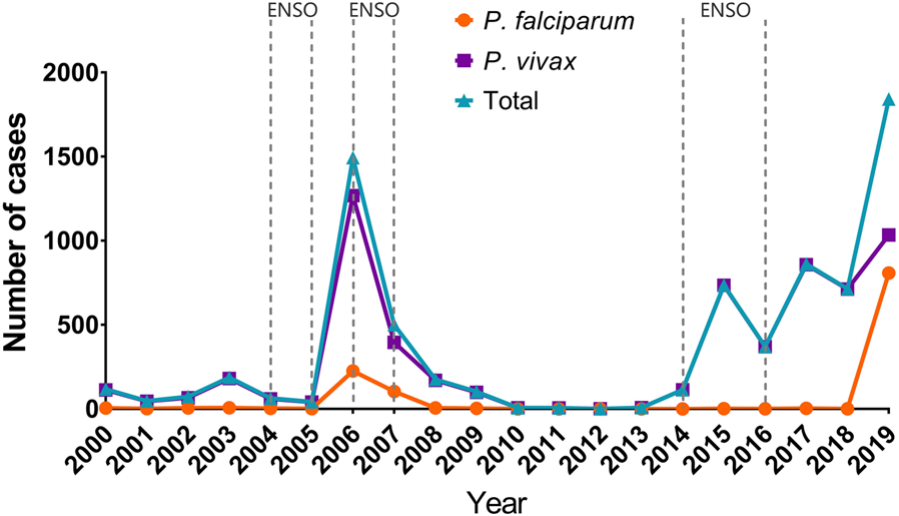
General trend of malaria cases in Condorcanqui, Amazonas Region, Peru. *Plasmodium falciparum* and *Plasmodium vivax* cases reported by the DIRESA-Amazonas in the past 19 years. El Niño–Southern Oscillation (*ENSO*) events are shown within dotted lines. The increase in the number of malaria cases at different time points is associated with these events

In the Americas, the population at risk of malaria is 138 million, with 753,700 confirmed cases and 338 deaths in 2018 [1]. The greatest number of cases is reported in the Amazon basin, and Peru represents 6% of the total cases in the Americas [1]. Between 2015 and 2019, a total of 244,723 malaria cases were notified in 22 regions of Peru. Most of these cases were reported in Loreto (95.6%), Amazonas (1.8%), Junín (1.0%), and San Martín (0.7%) [2]. Approximately, 95% of the cases in Amazonas are found in the Río Santiago District (Condorcanqui Province), an area where native communities live on the banks of the Santiago River, with no electricity, drinking water, or road access, having the rivers as the primary means of transportation [3]. Last year, Amazonas reported a 2.5-fold increase, with an unusual circulation of *Plasmodium falciparum*, representing a risk for severe malaria and mortality. It is worth noting that since 2015 only four *P. falciparum* imported cases were reported. In 2018, 705 malaria cases were reported and this number increased to 1843 in 2019, including 807 new *P. falciparum* cases (Fig. 1) [4]. Factors such as poor living conditions, movement of people, poor treatment compliance, climate change, the circulation of insecticide-resistant mosquitoes plus a more articulate malaria surveillance in this area could be responsible for these numbers. Moreover, people with malaria infections may remain asymptomatic for a long period of time, acting as carriers and/or reservoirs and therefore contributing to malaria transmission [5, 6]. In this context, early diagnosis and prompt treatment represent the main actions for malaria control and elimination.

Here, an epidemiological study was performed in eight sanitary districts of Rio Santiago, Condorcanqui, including follow-up of all positive cases until completion of treatment, and analysis of risk factors from positive malaria cases. Knowledge obtained from this report is useful to determine effective malaria control strategies and interrupt *P. falciparum* settlement in Amazonas.

## Methods

The Regional Directorate of Health-Amazonas (DIRESA-Amazonas) in collaboration with the Condorcanqui Health Network performed malaria active case detection (ACD) activities, in the Rio Santiago District, Condorcanqui Province, Amazonas Region of Peru, in response to the *P. falciparum* outbreak reported in 2019. The Institute of Tropical Diseases (IET) of the Universidad Nacional Toribio Rodríguez de Mendoza (UNTRM) collaborated with these institutions during ACDIII activities between January 31st and February 10th of 2020 and the data collected was analysed in this manuscript. Activities were performed at the community centres and a door-to-door strategy was adopted for those families who did not attend the open call. This study was performed as part of the surveillance activities approved by the DIRESA-Amazonas and the National Ministry of Health.

Informed consent was obtained from “Apus” (chiefs of the communities) as well as from all individual participants included in the study. For underage children, informed consent of parents or legal guardian was obtained.

### Study site description

**Fig. 2:**
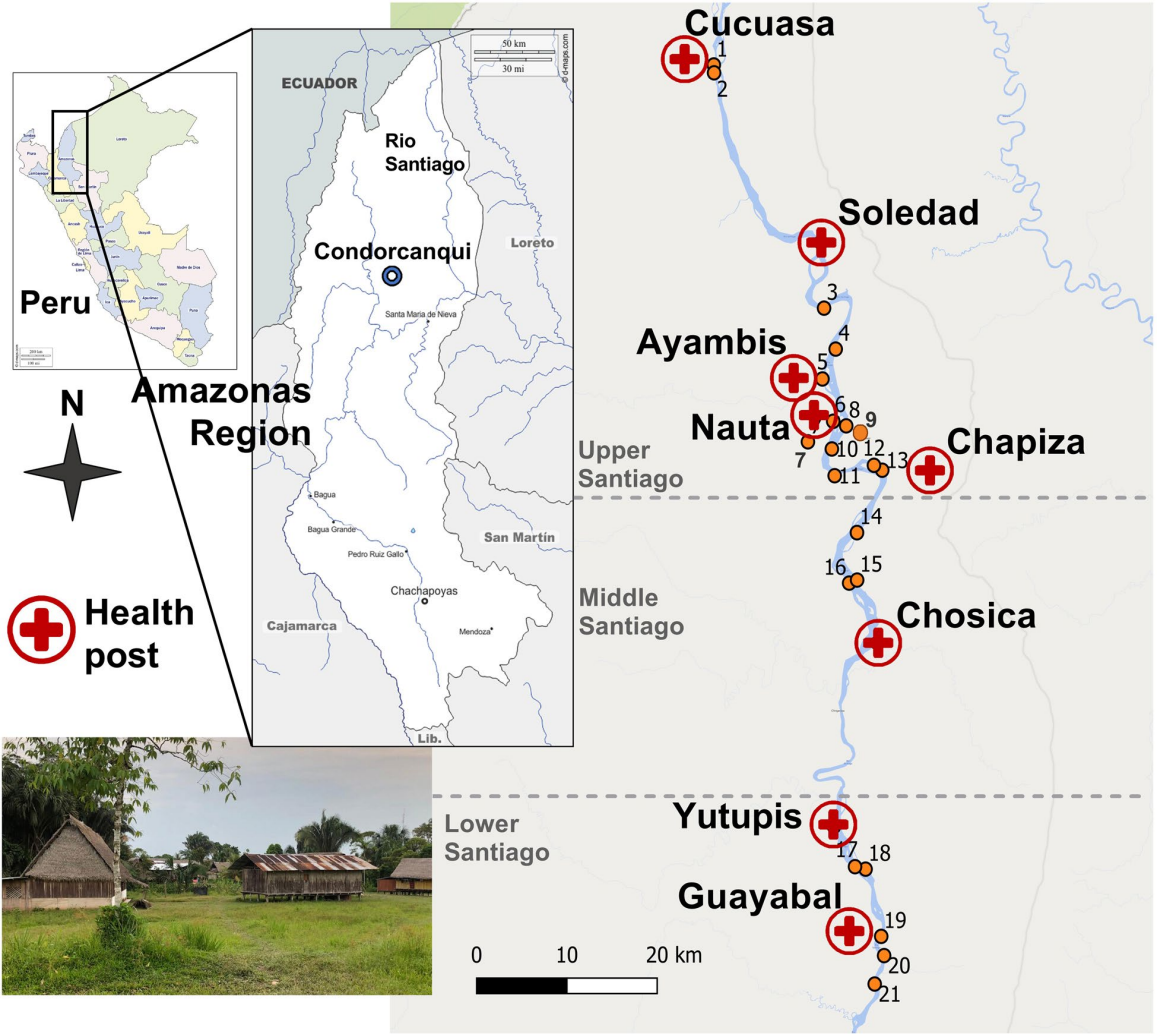
Map of collection sites in the Rio Santiago District, Condorcanqui Province, Amazonas Region, Peru. This map depicts a picture and the communities located along the river with their corresponding health posts and the sanitary districts. Cucuasa (1. Cucuasa, 2. Pachis), Soledad (3. Nueva Nazareth, 4. Palometa), Ayambis (5. Ayambis), Nauta (6. Nauta, 7. Tipishka, 8. Ajachim, 9. Waisram, 10. Porvenir), Chapiza (11. Alianza Progreso, 12. Isla, 13. Chapiza, 14. Nueva Esperanza), Chosica (15. Panguana, 16. Pampa Entsa), Yutupis (17. Camit Entsa, 18. Shebonal) and Guayabal (19. Guayabal, 20. Fortaleza, 21. Democracia)

Condorcanqui Province is located in the northern jungle of the Amazonas Region of Peru (Fig. 2), and it is part of the Marañon Basin. This province is bordered by the Republic of Ecuador to the North; by Loreto to the East, by Utcubamba and Bongara provinces to the South, and by Cajamarca to the West. Condorcanqui is divided in three districts: Nieva, El Cenepa, and Rio Santiago, and it has an extension of 17,892 km^2^ [3], with an estimated population of 42,470 [7].

The rugged relief of this region has an extensive fluvial network constituted by the Marañon River and its tributaries Cenepa, Nieva, and Santiago, in addition to a large number of streams of different flows and sizes. Condorcanqui has a humid tropic climate with temperatures that can reach 35 °C, annual average rainfall around 4800 mm, and relative humidity above 90%. The rainy season occurs between October to December, but it could last until May [3].

### Study design and data collection

A total of 2718 individuals (representing the 47.4% of the total population) were screened for malaria infections in 21 native communities. These communities were grouped in eight “sanitary districts” (Cucuasa, Soledad, Ayambis, Nauta, Chapiza, Chosica, Yutupis, and Guayabal) according to their corresponding health post (Fig. 2). The Rio Santiago District is divided into three major areas: Upper Santiago (Cucuasa, Soledad, Ayambis, Nauta, and Chapiza), Middle Santiago (Chosica), and Lower Santiago (Yutupis and Guayabal).

Blood samples were collected from all participants by finger pricks for preparation of thick and thin blood smears. Rapid diagnostic tests (RDTs) were available only for people reporting symptoms. Microscopy results were given the next day and all positive individuals were treated according to the Peruvian Ministry of Health guidelines for malaria cases [8].

A registry of socio-demographic variables such as the name of the community, age, gender, and presence of symptoms was recorded for coded positive malaria cases with no personal identifiers. Data of previous malaria cases in Condorcanqui was obtained from the DIRESA-Amazonas database with no names or personal identifiers.

### Diagnosis and treatment

Thick and thin blood smears were stained with Giemsa. The slides were examined under compound microscopes (Olympus) at 1000× magnification with oil to detect malaria parasites. Ring, trophozoite, or gametocyte stages were identified and assessed for parasite density using the semi-quantitative method according to the Peruvian Ministry of Health guidelines [8]. The microscopy team consisted of four field technicians, two with medium and two with high malaria expertise. The high expertise technicians confirmed the diagnosis. Additionally, all slides were independently read and confirmed at the Candungos or Galilea health centres. It is worth noting that 10% of malaria slides from all Peruvian national health centres undergo quality control at the National Institute of Health in Peru (INS) according to Ministerial Resolution N°. 461-2010/MINSA.

SD Bioline Malaria Ag P.f/P.f/P.v RDTs were available only for patients who presented symptoms. Fifteen percent of the positive samples (n = 35) were randomly collected in Whatman™ Protein Saver cards 903 and confirmed by qPCR at IET for quality control according to a previously reported protocol [9]. PET-PCR assays were run with 5 μl of DNA template using QuantStudio 5 thermocycler (Thermo Fisher, Waltham, MA, USA). As previously established, a CT value of < 40 was considered positive; samples with CT values above 40 were considered to be negative.

Positive *Plasmodium vivax* cases were treated with chloroquine and primaquine for 7 days and positive *P. falciparum* cases with mefloquine and artesunate for 3 days with additional primaquine (0.75 mg/kg) the last day of treatment. Mixed infections were treated with mefloquine, artesunate, and subsequently primaquine (0.5 mg/kg/day) for 7 days. Children under 12 years of age were treated according to their weight [8].

### Variable definitions and descriptive analysis

Malaria cases were classified into six age groups defined as: children under 5 years old, children (5–11 years old), teenagers (12–17 years old), youngsters (18–29 years old), adults (30–59 years old) and seniors (over 60 years old). Type of infection was recorded according to the *Plasmodium* species observed by microscopy; mixed infections were reported if both *P. vivax* and *P. falciparum* were present. Parasitaemia was classified as “low” if less than 40 parasites were found in at least 100 fields and “high” if more than 40 parasites were found. Symptomatic individuals were defined as those who presented any of the following symptoms at the time of taking the blood sample: fever, headache, body pain, shivering, nausea, vomiting, and/or abdominal pain. Asymptomatic individuals were defined as persons with confirmed infection showing no symptoms of infection or disease.

### Statistical analyses

Prevalence of malaria positive cases with a 95% confidence interval (CI) was calculated for each sanitary district and according to species. Omnibus test (Chi-square) was used to determine the association between location (sanitary districts) and presence of malaria [10]. Then, a generalized linear model with binomial distribution was used to evaluate specific associations. The outcome variables were measured on a binary scale, presence (Z = 1) or absence (Z = 0) of the infection, considering sanitary district as the predictor without intercept. To recognize specific differences between sanitary districts, 95% CI, and the probability of infection were calculated. The probability of infection was calculated from the estimated parameter with the exponential function. Fixed effects model odds ratio (OR) with corresponding 95% CI was used to analyse the magnitude of relative risk for an association between independent variables such as gender (female), age (all categories), type of infection (*P. falciparum*), and parasitaemia (low) with the dependent variables, symptomatology (asymptomatic), type of infection (*P. falciparum*) and parasitaemia (low).

Additional bivariate cross-tabulations Pearson Chi-Square analysis with bilateral asymptotical significance (p-value of Chi-Square statistic) and Wald test from logistic regression were performed to examine these associations with 95% confidence. All the statistical analyses were based on Agresti, 2007 [10], and were performed using the IBM SPSS Statistics Subscription and Rstudio.

### Definition of spatial clusters

A spatial analysis to detect clusters with high and low risk of malaria infection was conducted using the SaTScan software (version 9.6 64-bit). A Bernoulli model was applied to evaluate the spatial distribution in sanitary districts with positive malaria cases relative to the control group (negative cases). Clusters were detected with a maximum spatial cluster size of 50% of the population at risk [11] and a circular spatial window shape. SaTScan considers the null hypothesis that malaria infections are randomly distributed and compares the observed and expected number of infections inside and outside each circular window. The inference of isotonic clusters was based on a Standard Monte Carlo simulation with 9999 number of replications. SaTScan reports a p-value for all obtained clusters, as well as a relative risk which is the estimated risk within the cluster divided by the estimated risk outside the cluster. Significant low and high infection clusters (p < 0.05) were mapped using QGIS version 3.10.8 for total malaria cases and per type of infection (*P. falciparum* and *P. vivax*). To analyse the effect of the surveillance in these communities, heat maps of malaria incidence per 1000 habitants, considering only passive surveillance before and after ACD were also included.

## Results

### Origin of the *P. falciparum* outbreak (2019–2020)

**Fig. 3:**
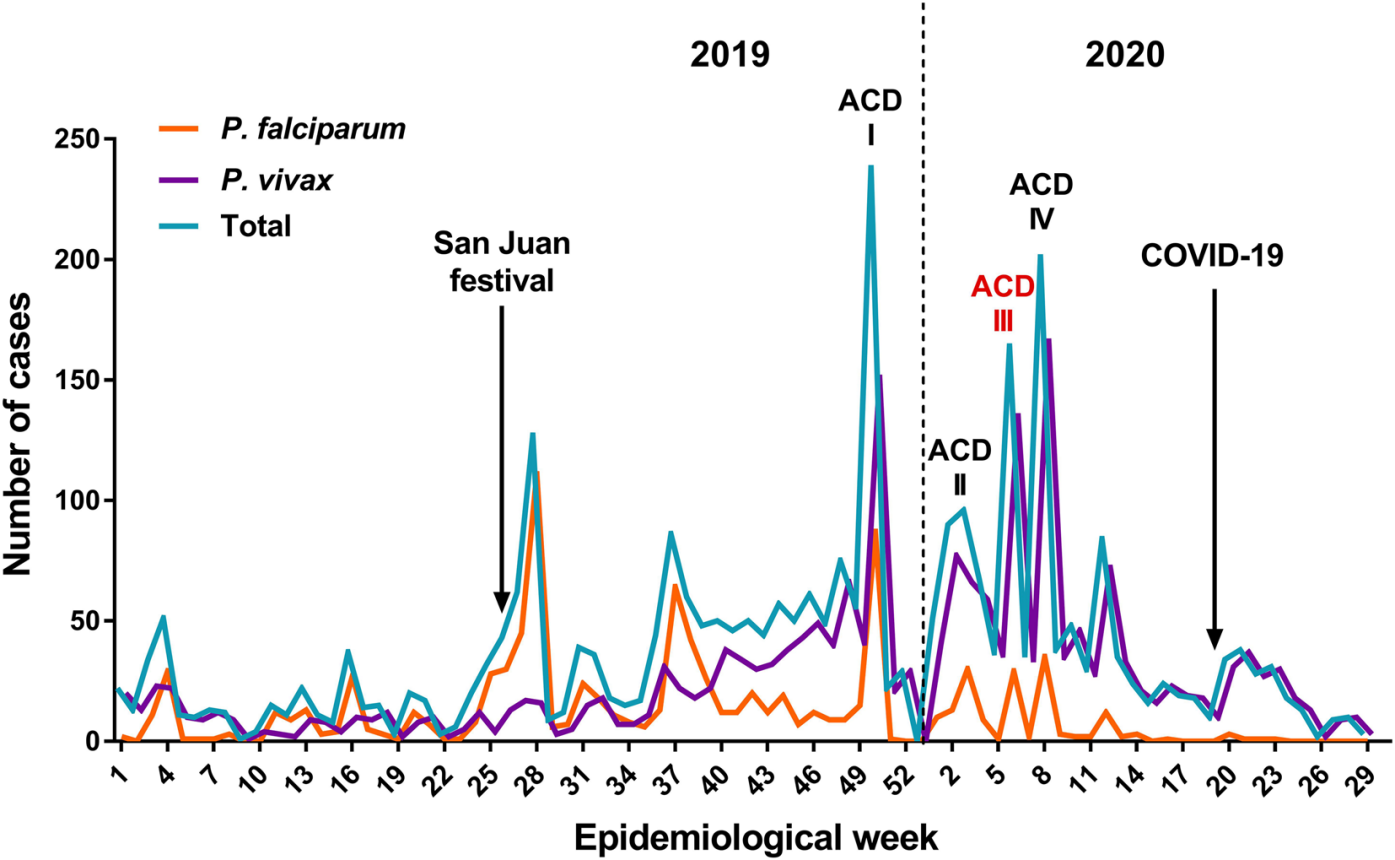
Malaria cases during 2019 and 2020 in Condorcanqui. Major events such as the San Juan festival and ACDs (I, II, III, and IV) are indicated. First COVID-19 reports in Condorcanqui are also indicated. Results from the following study correspond to ACD III (between January 31st and February 10th of 2020). *ACD* Active case detection

From 2015 to 2018, only four imported cases of *P. falciparum* were reported in Condorcanqui. The last case reported was a man visiting from the Shinguito community of Loreto (57 km away from Condorcanqui). Five months later, in January of 2019, the first two *P. falciparum* autochthonous cases were found in Soledad, a 3-year-old girl, and an 11-year-old boy. Then, sporadic cases were reported in the sanitary districts of Ayambis, Soledad, Chapiza, Nauta, Cucuasa, and Chosica. However, an unusual increase in the number of *P. falciparum* cases was notified after epidemiological week 25 of 2019 (Fig. 3, Additional file 1: Fig. S1) with clusters of cases in Chapiza, Chosica, and Nauta. After that, the cumulative cases (2019–2020) of *P. falciparum* increased up to 968 until epidemiological week 29 of 2020.

### Active case detection III results

#### Prevalence of malaria

A total of 220 malaria positive cases were found in the population screened by microscopy (n = 2718, 47.4%). The prevalence of malaria in this area was 8.1%, from which Chosica (13.2%) and Yutupis (13.4%) depicted the highest prevalence (Table 1). In most districts, *P. vivax* was higher than *P. falciparum*; except in Ayambis (6.2%) and Soledad (5.2%) where *P. falciparum* infections were greater. The highest *P. vivax* prevalence was found in Yutupis (13.4%), followed by Chosica (12.1%), Chapiza (7.2%), and Nauta (6.8%). Also, 3.6% of the total malaria positive cases (eight individuals) were *P. falciparum* gametocytes carriers, and only one of them showed symptoms.

**Table 1:** Prevalence of positive malaria cases per sanitary district from Río Santiago during the ACD III

Sanitary district^a^	Total population	Sampling (%)	Positive cases	Prevalence %	CI %	*P. vivax*		*P. falciparum*	
						Prevalence %	CI %	Prevalence %	CI %
Cucuasa (2)	454	315 (69.4%)	1	0.3	(0.00–0.94)	0.3	(0.00–0.94)	0	(0.00–0.00)
Soledad (2)	265	135 (50.9%)	10	7.4	(2.99–11.83)	2.2	(0.00–4.71)	5.2	(1.44–8.93)
Ayambis (1)	350	241 (68.9%)	18	7.5	(4.15–10.79)	0.8	(0.00–1.98)	6.2	(3.17–9.27)
Nauta (5)	790	366 (46.3%)	34	9.3	(6.32–12.26)	6.8	(4.25–9.42)	1.6	(0.34–2.94)
Chapiza (4)	2340	741 (31.7%)	70	9.4	(7.34–11.55)	7.2	(5.30–9.01)	2	(1.01–3.04)
Chosica (2)	391	272 (69.6%)	36	13.2	(9.21–17.26)	12.1	(8.25–16.01)	1.1	(0.00–2.34)
Yutupis (2)	460	328 (71.3%)	44	13.4	(9.73–17.10)	13.4	(9.73–17.10)	0	(0.00–0.00)
Guayabal (3)	680	320 (47.1%)	7	2.2	(0.58–3.79)	2.2	(0.58–3.79)	0	(0.00–0.00)
Total (21)	5730	2718 (47.4%)	220	8.1	(7.07–9.12)	6.2	(5.28–7.09)	1.7	(1.21–2.18)

Mixed infections prevalence 0.2%

CI: 95% confidence level

^a^Number of communities are shown in parentheses

### qPCR results

From the 35 positive samples collected for qPCR confirmation, 10 were *P. falciparum* and 25 were *P. vivax*. These results have 100% agreement with the ones obtained by microscopy.

### Spatial cluster analysis

**Fig. 4:**
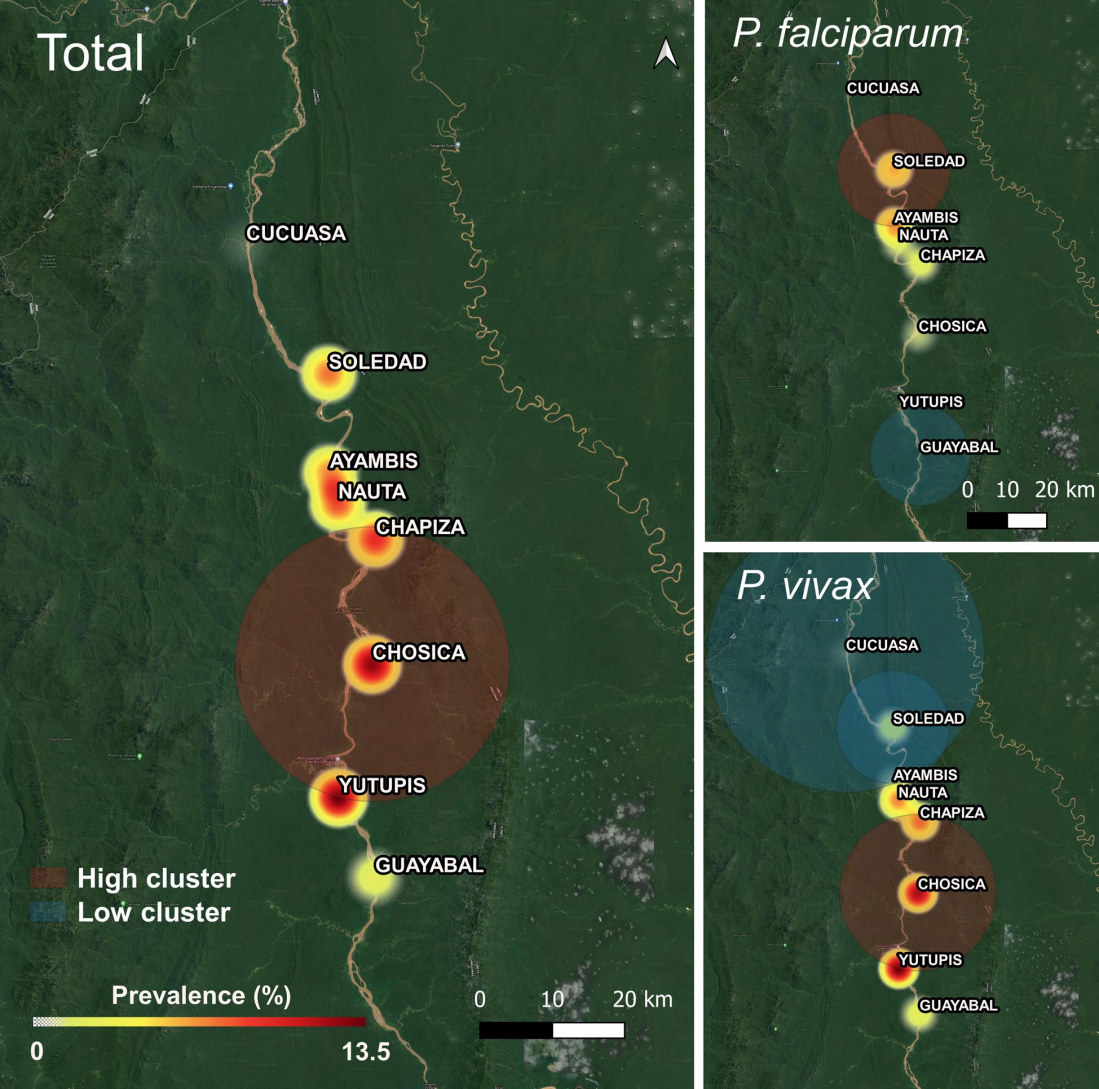
Spatial clustering of malaria cases in Rio Santiago during the ACD III. Heat map of malaria prevalence (%) and spatial clustering. Total malaria cases (left), *Plasmodium falciparum* cases (upper right), and *Plasmodium vivax* (lower right). Maximum value: 13.5%

SaTScan analysis detected a cluster of high malaria infection risk in the Middle part of Rio Santiago including Chapiza, Chosica, and Yutupis (Table 2, Fig. 4). Meanwhile, a high *P. falciparum* infection risk cluster was found in Soledad and Ayambis from Upper Santiago and a low relative risk cluster in Yutupis and Guayabal from Lower Santiago. *Plasmodium vivax* showed at least three clusters, a high-risk cluster corresponding to Chosica, Chapiza, and Yutupis and two other low-risk clusters from Upper Santiago.

**Table 2:** Spatial cluster analysis of malaria cases in Rio Santiago during the ACD III

Type of infection	High/low cluster^a^	Radius (km)	Sanitary districts	Obs	Exp	RR	p
*P. falciparum*	High cluster	14.21	Soledad, Ayambis	22	7	5.39	0.0001
*P. falciparum*	Low cluster	12.57	Yutupis, Guayabal	0	11	0	0.0001
*P. vivax*	High cluster	19.69	Chosica, Chapiza, Yutupis	130	83	3.48	0.0001
*P. vivax*	Low cluster	14.21	Soledad, Ayambis	5	22	0.2	0.0002
*P. vivax*	Low cluster	34.83	Cucuasa, Soledad Ayambis	6	42	0.11	0.0001
Total	High cluster	19.69	Chosica, Chapiza, Yutupis	150	109	2.2	0.0001

*Obs* observed cases, *Exp* expected cases, *RR* Relative risk

^a^Only significant clusters are shown

### Sociodemographic characteristics

From all positive cases, 76.4% were diagnosed with *P. vivax*, 20.9% with *P. falciparum* and 2.7% were mixed infections. In general, there were more positive females (58.2%) than males (41.8%) and according to the age distribution, the number of malaria cases was higher in children between 5 and 11 years old (30.5%) (Table 3). In addition, 59.5% (n = 131) of infected people were asymptomatic, from which 16.8% (n = 37) were *P. falciparum* and 42.3% (n = 93) were *P. vivax*. Only one asymptomatic mixed infection was reported. However, it is worth noting that from all *P. falciparum* cases, 80.4% reported no symptoms compared to 55.4% of *P. vivax* cases.

**Table 3:** Characteristics of the positive malaria cases (N = 220) in Río Santiago during the ACD III

	*Plasmodium falciparum*				*Plasmodium vivax*				Mixed infection				Total	
	Asymptomatic		Symptomatic		Asymptomatic		Symptomatic		Asymptomatic		Symptomatic			
	N	%	N	%	N	%	N	%	N	%	N	%	N	%
Age group														
Children under 5	5	13.5	1	11.1	18	19.4	11	14.7	0	0.0	0	0.0	35	15.9
Children (5–11)	12	32.4	2	22.2	26	28.0	24	32.0	0	0.0	3	60.0	67	30.5
Teenagers (12–17)	3	8.1	2	22.2	21	22.6	16	21.3	0	0.0	1	20.0	43	19.5
Youngsters (18–29)	2	5.4	1	11.1	13	14.0	15	20.0	1	100.0	0	0.0	32	14.5
Adults (30–59)	14	37.8	3	33.3	14	15.1	7	9.3	0	0.0	1	20.0	39	17.7
Seniors (> 60)	1	2.7	0	0.0	1	1.1	2	2.7	0	0.0	0	0.0	4	1.8
Gender														
Female	22	59.5	9	100.0	53	57.0	41	54.7	0	0.0	3	60.0	128	58.2
Male	15	40.5	0	0.0	40	43.0	34	45.3	1	100.0	2	40.0	92	41.8
Parasitaemia														
High	16	43.2	4	44.4	51	54.8	63	84.0	1	100.0	5	100.0	140	63.6
Low	21	56.8	5	55.6	42	45.2	12	16.0	0	0.0	0	0.0	80	36.4
Total	37	80.4	9	19.6	93	55.4	75	44.6	1	16.7	5	83.3	220	100.0

### Associations and risk factors

According to the Omnibus test, there was a significant association between sanitary districts and malaria infections (χ^2^ = 2326.74, p < 0.001). The estimated parameter of the generalized linear model with binomial distribution indicated significant differences among all sanitary districts (p < 0.001) with a higher probability of infection in Ayambis, Nauta, Chapiza, Chosica, and Yutupis compared to Cucuasa and Guayabal (Table 4).

**Table 4:** Estimated parameter of the generalized linear model and probability of infection per sanitary district during the ACD III

Sanitary district	Estimated parameter	(95% CI)	p	Probability
Cucuasa	− 5.749	(− 7.712 to − 3.786)	< 0.001	0.003
Soledad	− 2.526	(− 3.170 to − 1.882)	< 0.001	0.080
Ayambis	− 2.517	(− 2.997 to − 2.037)	< 0.001	0.081
Nauta	− 2.279	(− 2.632 to − 1.926)	< 0.001	0.102
Chapiza	− 2.260	(− 2.506 to − 2.014)	< 0.001	0.104
Chosica	− 1.880	(− 2.231 to − 1.530)	< 0.001	0.153
Yutupis	− 1.865	(− 2.182 to − 1.547)	< 0.001	0.155
Guayabal	− 3.800	(− 4.549 to − 3.051)	< 0.001	0.022

Generalized linear model for malaria cases with binomial distribution

On the other hand, the analysis of risk factors showed significant associations between low parasitaemia and asymptomatic cases (OR = 3.71, 95% CI 1.97–6.98, p < 0.001) and between *P. falciparum* infections and asymptomatic cases (OR = 3.32 95% CI 1.51–7.30, p = 0.003). Furthermore, significant associations between adults and *P. falciparum* infections (OR = 4.10 95% CI 1.93–8.72, p < 0.001), and between low parasitaemia and *P. falciparum* infections (OR = 2.74 95% CI 1.41–5.35, p = 0.003) were also found (Table 5). These results agree with the Pearson chi-square analysis outcome (Additional file 2: Table S1).

**Table 5:** Fixed effects of univariate logistic regression models of symptomatology and type of infection

Independent variable (predictor)	Symptomatology (Asymptomatic)			Type of infection *(P. falciparum*)			Parasitaemia (Low)		
	OR	(95% CI)	p	OR	(95% CI)	p	OR	(95% CI)	p
Gender (female)	0.91	(0.53–1.57)	0.735	1.63	(0.82–3.24)	0.165	1.55	(0.88–2.75)	0.132
Age (children under 5)	1.37	(0.64–2.91)	0.419	0.72	(0.28–1.85)	0.495	0.85	(0.40–1.82)	0.679
Age (children)	0.85	(0.47–1.51)	0.572	1.03	(0.51–2.10)	0.930	0.83	(0.45–1.53)	0.553
Age (teenagers)	0.83	(0.42–1.62)	0.579	0.43	(0.16–1.17)	0.099	1.04	(0.52–2.08)	0.915
Age (youngsters)	0.63	(0.30–1.35)	0.237	0.35	(0.10–1.20)	0.096	0.91	(0.41–2.01)	0.813
Age (adults)	1.93	(0.90–4.11)	0.089	*4.10*	(*1.93–8.72*)	*< 0.001*	1.65	(0.82–3.36)	0.163
Age (seniors)	0.67	(0.09–4.88)	0.696	1.22	(0.12–12.03)	0.864	0.55	(0.06–5.41)	0.610
Parasitaemia (low)	*3.71*	(*1.97–6.98*)	*< 0.001*	*2.74*	(*1.41–5.35*)	*0.003*	–	–	–
Type of infection (*P. falciparum*)	*3.32*	(*1.51–7.30*)	*0.003*	–	–	–	–	–	–

Numbers in italics represent significant values

Children under 5, children (6–11), teenagers (12–17), youngsters (18–29), adults (30–59), and seniors (> 60)

*OR* odds ratio

## Discussion

Malaria disease caused by *P. vivax* and *P. falciparum* represents a major public health problem in Peru. Most of the control efforts are focused in Loreto, where 95.6% of all cases are reported [2]. However, since 2018, Amazonas is the Peruvian region with the highest incidence of cases, affecting native communities, such as the ones living on the banks of the Santiago River. Thus, understanding the distribution and risk factors associated with malaria infections in native communities is important to strengthen the malaria control programmes with targeted activities. Condorcanqui province has shown increasing reported *P. vivax* cases during the past 2 years with the recent emergence of *P. falciparum* cases in 2019 [4]. In the epidemiological week 29 of 2018, only one *P. falciparum* imported case from Shinguito, Loreto, was reported. This community is 57 km apart from the community of Soledad in Condorcanqui, where the first two *P. falciparum* autochthonous cases were reported. These cases corresponded to symptomatic children that were taken to the health post; however, it is possible that infected adults did not report symptoms and remained unattended, contributing to the transmission of the disease. The following weeks, *P. falciparum* cases spread to Soledad neighbouring communities and other sanitary districts. Towards the epidemiological week 27 of 2019, a high number of cases was observed. This fact can be explained by the increase of travels to and from Loreto due to The San Juan Festival, which is a major event in the Amazon of Peru. This festival is held for one week with the main celebration date on June 24th, where people party and exchange goods. This could have increased the number of infections and the spread of *P. falciparum* to other communities such as Chapiza, Nauta, and Chosica. Moreover, Alianza Progreso, located within the sanitary district of Chapiza, is a centre of several social, commercial, and economical activities, where people from other communities stay after 6 pm (time when female *Anopheles* mosquitoes usually take their blood meal), representing a hotspot for malaria transmission. Besides, climate changes caused by El Niño-Southern Oscillation could be related to the reemergence of malaria in this area over the years. Since the number of malaria cases increased in Condorcanqui, DIRESA-Amazonas organized several ACDs to control malaria and the spread of *P. falciparum* to other native communities.

According to previous data from ACD I and II, the highest prevalence of malaria was reported in Chosica (19.1%), followed by Nauta (12.4%) and Chapiza (10.2%) [4]. In this study (ACD III), Yutupis (13.4%) and Chosica (13.2%) reported the highest number of cases. Furthermore, a high-risk cluster for malaria transmission, included the sanitary districts of Chapiza, Chosica, and Yutupis, indicating that the cases were also spreading to other communities southern to Chosica (Fig. 4). Additionally, *P. falciparum* and *P. vivax* presented different spatial distribution. The recent introduction of *P. falciparum* in Soledad can explain the presence of a high-risk cluster, while *P. vivax* is endemic in all Rio Santiago. Moreover, six mixed infections were reported, which represented a challenge for a correct diagnosis and treatment of patients.

A previous study in riverine communities of Loreto determined that malaria prevalence and the micro-geographical heterogeneity of *P. vivax* parasitaemia are linked to specific factors such as travel and occupation, which created a complex transmission dynamics in these communities [12]. These are important factors to consider when analysing trends in malaria prevalence along with environmental conditions that could facilitate the establishment of new mosquito breeding sites.

In Condorcanqui, descriptive analysis of positive cases exhibited a higher number in females than in males and the number of cases in children (5–11 years old) was also higher than in other age groups. The lack of complete sociodemographic data of negative cases hinders further statistical analysis of infection risk. Although a door-to-door strategy was considered for individuals who did not attend the open call, some men were working in the field and some declined their participation in the study which might generated a bias towards women participants.

Nevertheless, it has been reported that in malaria-endemic zones, children and pregnant women are the groups at highest risk and the most affected due to poor socio-economic conditions and nutritional problems [12], something that is also characteristic of these communities, given the high rates of anemia in the population. Anaemia in Peru is a long-standing problem, in 2018 the prevalence in children (6 months to 3 years old) was 43.5%, being even higher in the rural jungle (53.5%) [13]. Moreover, specific micronutrients play key roles in the function of the immune system and certain deficiencies could predispose to malaria infections [14, 15]. Hence, it is important to focus efforts on both risk groups for an efficient malaria control program.

On the other hand, although *P. vivax* cases were predominant, most *P. falciparum* cases in Condorcanqui were asymptomatic. Asymptomatic malaria infections hamper disease control. Under normal circumstances, these patients do not seek treatment and they become parasite reservoirs contributing to the malaria cycle in the population [16].

Additionally, the evaluation of different risk factors showed significant associations between asymptomatic cases and low parasitaemia, these relative risks are in agreement with previous reports of endemic areas [17, 18], suggesting that the population is acquiring immunity and there is a risk of falciparum malaria becoming endemic. Moreover, significant associations were also found between *P. falciparum,* asymptomatic cases, and adults (30–59 years old), consistent with previous studies [17, 19–21]. The higher relative risk of presenting *P. falciparum* infection in adults may be due to their activities, such as farming, hunting, or fishing, being in close contact with mosquito breeding sites, and more exposed to infections than other age groups. Low parasitaemia cases represent a challenge to the correct diagnosis and according to previous studies, there are significant numbers of submicroscopic asymptomatic cases only detectable by qPCR given that these subjects retained control over the parasitaemia [22]. It is worth noting that asymptomatic malaria cases reported in this study could be underestimated given that qPCR was not performed for all samples; however, quality control of the samples showed consistent results for both techniques. Also, an active report of gametocyte density is important considering that they are responsible for the continuity of malaria transmission [11]. Moreover, there is concern that infected individuals carrying gametocytes do not seek treatment if they experience no symptoms [16].

Rapid diagnostic tests (RDTs) could be considered a complementary tool for prompt malaria diagnosis [23], especially in a field setting; however, it is important to consider that *Pfhrp2* and *Pfhrp3* deletions have been previously reported in Peru [24] and these could affect the efficiency of RDTs for *P. falciparum* detection. Thus, it is important to improve malaria diagnosis in native communities of Condorcanqui using complementary strategies, such as microscopy and RDTs or molecular biology techniques, for an effective malaria control [25].

Despite limited access to health services, completion of *P. falciparum* treatment in 3 days is achievable compared to *P. vivax* treatment, which requires supervision for 7 days [8]. Furthermore, *P. vivax* hypnozoites could be responsible for recurrences within 24 weeks [26], which represents a problem for *P. vivax* control. Comparison of *P. falciparum* and *P. vivax* cases detected during passive surveillance before and after the four ACDs showed a significant decrease in *P. falciparum* prevalence, indicating a successful control of the outbreak; while *P. vivax* hotspots continued to be present (Additional file 3: Fig. S2, Additional file 4: Fig. S3).

Although the number of malaria cases decreased after epidemiological week 20 of 2020, it has to be considered that the COVID-19 pandemic has heavily impacted the health services and the report systems of other diseases. The first COVID-19 case was reported in Condorcanqui towards the epidemiological week 19 of 2020, and by week 23, cases spread to other sanitary districts, reporting over 500 cases. It is possible that malaria infections are currently underestimated and that the increase of COVID-19 cases hinders not only *P. falciparum* but also *P. vivax* control strategies. This represents a serious health problem given that malaria and COVID-19 could become syndemics (synergistic epidemics). Continue surveillance of malaria in endemic regions is important to prevent severe malaria and deaths as a result of a co-infection with SARS-CoV-2.

## Conclusion

This article represents the first study describing the epidemiology of malaria cases in native communities of Condorcanqui, Amazonas, the first reported *P. falciparum* outbreak, and the presence of asymptomatic cases. Results presented here are useful to understand malaria transmission in native communities and allow targeted activities for malaria surveillance and control in this area. In conclusion, it is important to improve diagnosis to detect as many asymptomatic and symptomatic cases as possible to promptly treat and prevent the spread of this disease to other communities of Amazonas, Peru. Moreover, the current malaria situation in Condorcanqui is worrying, since lack of follow-up can lead to the establishment of *P. falciparum* and syndemics with COVID-19.

## Supplementary Information

**Additional file 1: Fig. S1.** Malaria cases per sanitary district during 2019 and 2020. Major events such as the San Juan festival and ACDs (I, II, III, and IV) are indicated. First COVID-19 reports in Condorcanqui are also indicated. **a.** *Plasmodium falciparum* cases. **b.** *Plasmodium vivax* cases.

**Additional file 2: Table S1.** Pearson chi-square (χ^2^) and contingency coefficient for positive malaria cases in Río Santiago.

**Additional file 3: Fig. S2.** Heat map of *Plasmodium falciparum* incidence per 1000 habitants before and after all ACD. Maps show 21 weeks before ACD I (left) and 21 weeks after ACD IV (right). Maximum value: 254.

**Additional file 4: Fig. S3.** Heat map of *Plasmodium vivax* incidence per 1,000 habitants before and after all ACD. Maps show 21 weeks before ACD I (left) and 21 weeks after ACD IV (right). Maximum value: 254.

## Data Availability

The datasets during and/or analysed during the current study available from the corresponding author on reasonable request.

## Abbreviation

DIRESA: Regional Directorate of Health
