# Supplementary figures and images for: Plasmodium falciparum outbreak in native communities of Condorcanqui, Amazonas, Perú
Source: Malar J. 2021 Feb 12;20:88. doi: 10.1186/s12936-021-03608-2 (PMC7880654; doi:10.1186/s12936-021-03608-2)

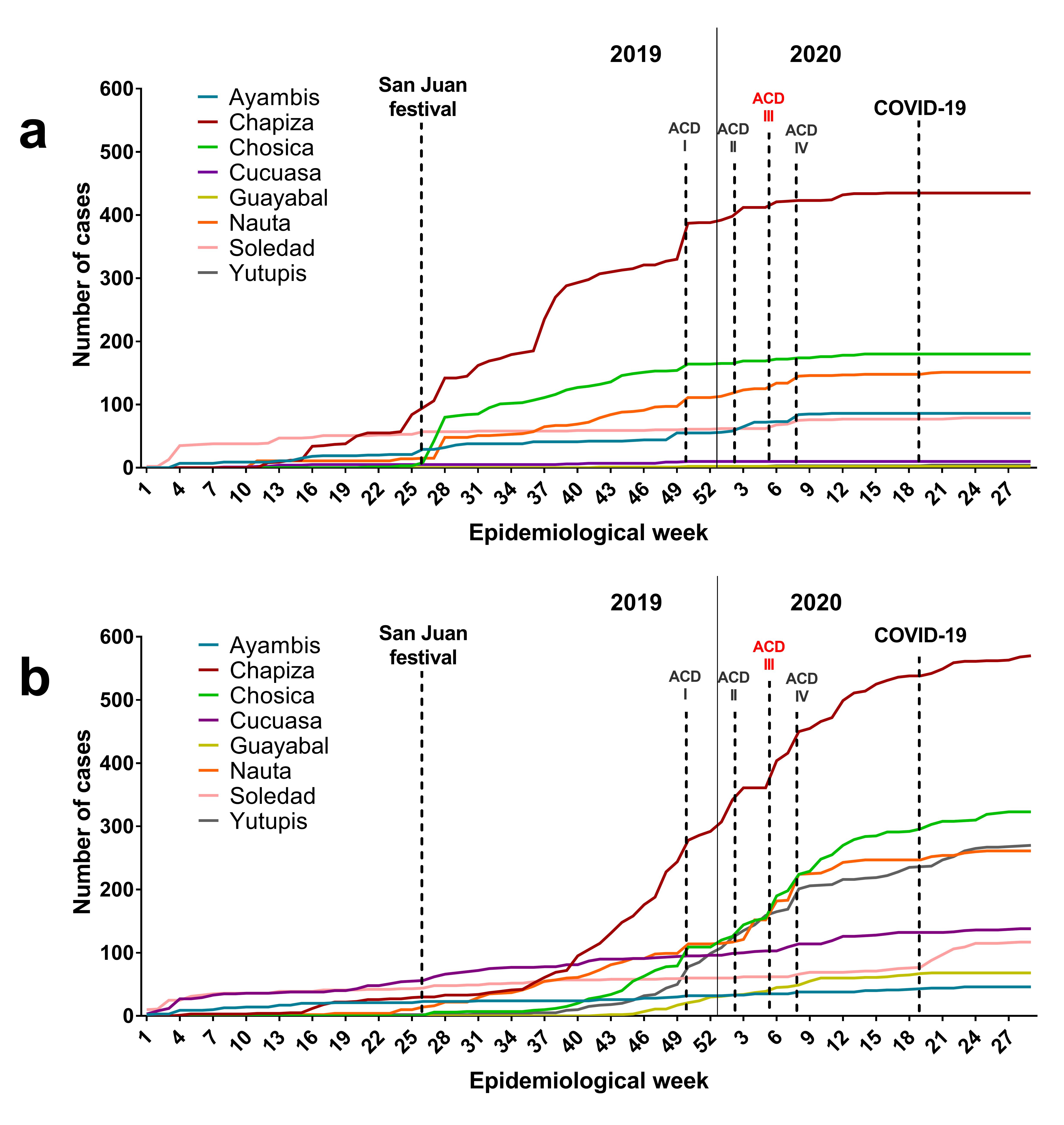

Supplement: Supplementary file 1 — Additional file 1: Fig. S1. Malaria cases per sanitary district during 2019 and 2020. Major events such as the San Juan festival and ACDs (I, II, III, and IV) are indicated. First COVID-19 reports in Condorcanqui are also indicated. a. Plasmodium falciparum cases. b. Plasmodium vivax cases. [file 12936_2021_3608_MOESM1_ESM.jpg]

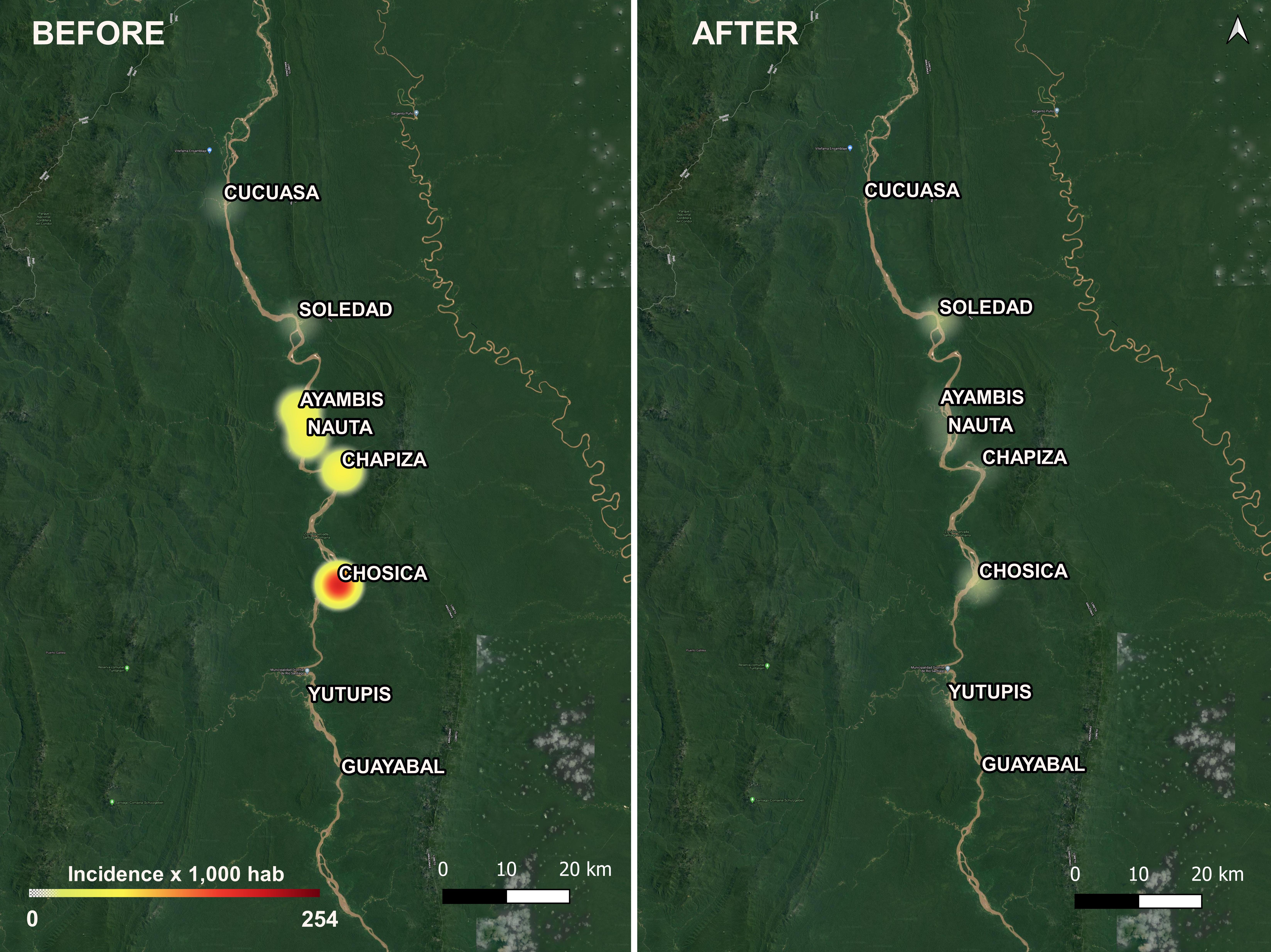

Supplement: Supplementary file 3 — Additional file 3: Fig. S2. Heat map of Plasmodium falciparum incidence per 1000 habitants before and after all ACD. Maps show 21 weeks before ACD I (left) and 21 weeks after ACD IV (right). Maximum value: 254. [file 12936_2021_3608_MOESM3_ESM.jpg]

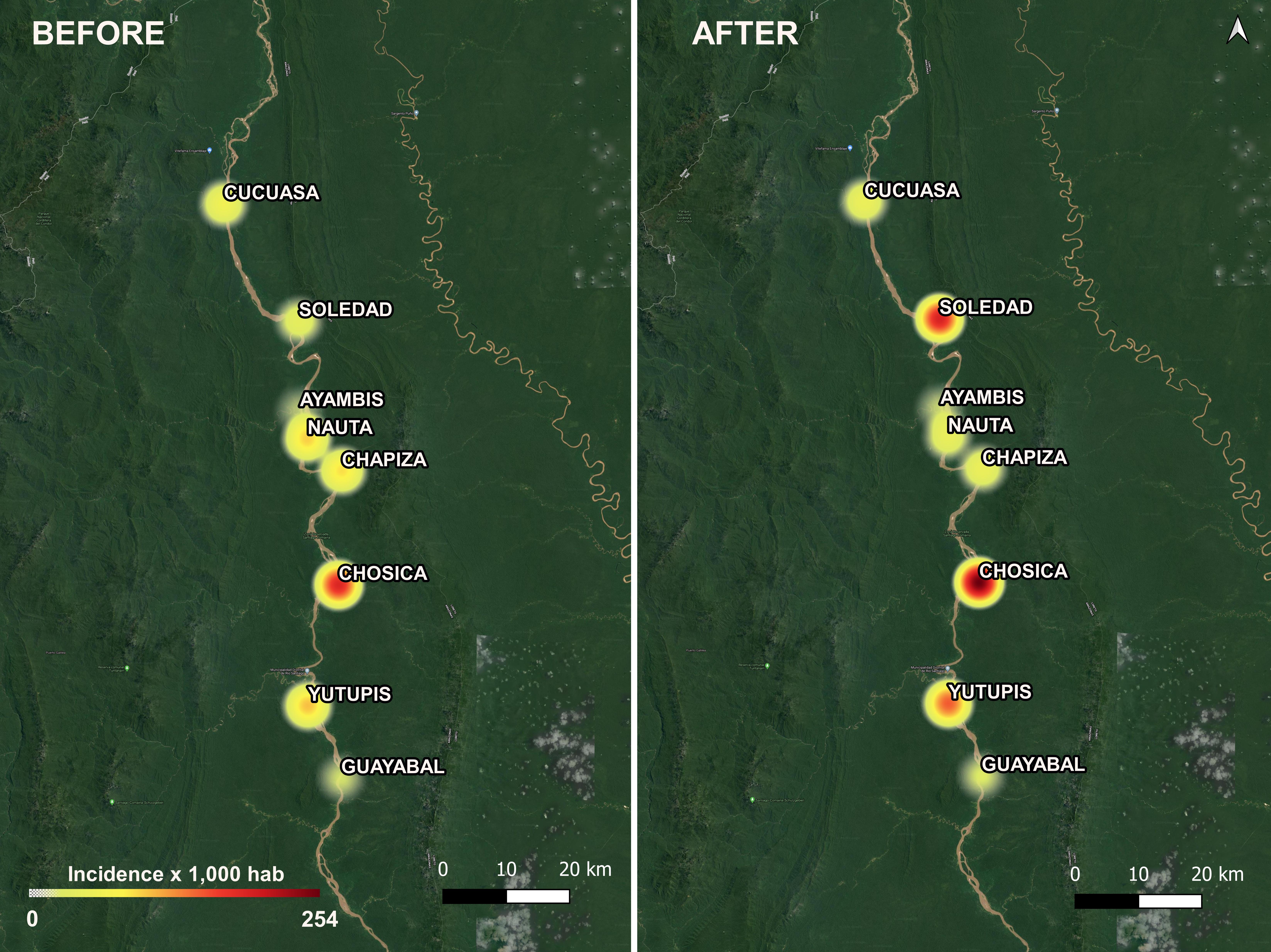

Supplement: Supplementary file 4 — Additional file 4: Fig. S3. Heat map of Plasmodium vivax incidence per 1,000 habitants before and after all ACD. Maps show 21 weeks before ACD I (left) and 21 weeks after ACD IV (right). Maximum value: 254. [file 12936_2021_3608_MOESM4_ESM.jpg]
