# Supplementary material for: Plasmodium falciparum outbreak in native communities of Condorcanqui, Amazonas, Perú
Source: Malar J. 2021 Feb 12;20:88. doi: 10.1186/s12936-021-03608-2 (PMC7880654; doi:10.1186/s12936-021-03608-2)
Supplement: Supplementary file 2 — Additional file 2: Table S1. Pearson chi-square (χ2) and contingency coefficient for positive malaria cases in Río Santiago. [file 12936_2021_3608_MOESM2_ESM.docx]

**Table S1. Pearson chi-square (χ2) and contingency coefficient for positive malaria cases in Río Santiago.**

| Independent variable (predictor) | Symptomatology (Asymptomatic) | | | Type of infection  (*P. falciparum*) | | |
| --- | --- | --- | --- | --- | --- | --- |
|  | **χ2** | **Contingency coefficient** | **p** | **χ2** | **Contingency coefficient** | **p** |
| Gender (Female) | 0.12 | 0.023 | 0.734 | 1.95 | 0.095 | 0.163 |
| Age (Children) | 0.01 | 0.005 | 0.942 | 0.18 | 0.029 | 0.669 |
| Age (Teenagers) | 0.31 | 0.037 | 0.578 | 2.85 | 0.115 | 0.091 |
| Age (Youngsters) | 1.42 | 0.08 | 0.234 | 3.00 | 0.118 | 0.083 |
| Age (Adults) | 2.95 | 0.115 | 0.086 | **14.79** | **0.254** | **<0.001** |
| Age (Seniors) | 0.15 | 0.026 | 0.695 | 0.03 | 0.012 | 0.863 |
| Type of infection (*P. falciparum*) | **9.52** | **0.206** | **0.002** | - | - | - |
| Parasitaemia (Low) | **17.36** | **0.274** | **<0.001** | **9.17** | **0.203** | **0.002** |
